# Metabolomic and transcriptomic changes underlying cold and anaerobic stresses after storage of table grapes

**DOI:** 10.1038/s41598-019-39253-8

**Published:** 2019-02-27

**Authors:** Itay Maoz, Mirko De Rosso, Tatiana Kaplunov, Antonio Dalla Vedova, Noa Sela, Riccardo Flamini, Efraim Lewinsohn, Amnon Lichter

**Affiliations:** 1 Department of Postharvest Science, Agricultural Research Organization (ARO)–the Volcani Center, HaMaccabim Road 68, P.O. Box 15159, Rishon LeZion, 7528809 Israel; 2 Department of Vegetable Crops, ARO–Newe Ya’ar Research Center, P.O. Box 1021, Ramat Yishay, 30095 Israel; 3 Robert H. Smith Faculty of Agriculture, Food and Environment, the Hebrew University of Jerusalem, Rehovot, 76100 Israel; 4 Council for Agricultural Research and Economics–Viticulture & Oenology (CREA-VIT), Laboratorio Chimico, Viale XXVIII Aprile 26, 31015 Conegliano, TV Italy; 5 Department of Plant Pathology and Weed Research, Agricultural Research Organization (ARO)–the Volcani Center, HaMaccabim Road 68, P.O. Box 15159, Rishon LeZion, 7528809 Israel

## Abstract

The currently accepted paradigm is that fruits and vegetables should be consumed fresh and that their quality deteriorates during storage; however, there are indications that some metabolic properties can, in fact, be improved. We examined the effects of low temperature and high-CO_2_ conditions on table grapes, *Vitis vinifera* L. cv. ‘Superior Seedless’. Berries were sampled at harvest (T0) and after low-temperature storage for 6 weeks under either normal atmosphere conditions (TC) or under an O_2_ level of 5 kPa and elevated CO_2_ levels of 5, 10 or 15 kPa (T5, T10, T15). Accumulation of 10 stilbenes, including *E*-ε-viniferin, *E*-miyabenol C and piceatannol, significantly increased under TC treatment as compared to T0 or T15. Sensory analysis demonstrated that elevated CO_2_ elicited dose-dependent off-flavor accumulation. These changes were accompanied by an accumulation of 12 volatile metabolites, e.g., ethyl acetate and diacetyl, that imparted disagreeable flavors to fresh fruit. Transcriptome analysis revealed enrichment of genes involved in pyruvate metabolism and the phenylpropanoid pathway. One of the transcription factors induced at low temperature but not under high CO_2_ was *VvMYB14*, which regulates stilbene biosynthesis. Our findings reveal the potential to alter the levels of targeted metabolites in stored produce through understanding the effects of postharvest treatments.

## Introduction

A large proportion of fresh produce is stored and transported from production sites to markets. This has been made possible by technological developments and intensive postharvest research that facilitate long-term storage with minimal losses and quality impairment^1^. The basic postharvest technology uses cold storage to reduce respiration rate and decay; freshness is preserved mainly by packaging to retain humidity around the produce. Another level of protection, termed modified atmosphere (MA), is achieved by packaging produce in bags designed to maintain higher CO_2_ and lower O_2_ levels during postharvest respiration. In a controlled atmosphere (CA), CO_2_ and O_2_ contents are set to optimal levels in a closed environment. These optimal levels are calculated to ensure maximal quality, minimal decay, and minimal off-flavors that may be generated by the anaerobic respiration that results from O_2_ reduction and CO_2_ enhancement.

Grape (*Vitis vinifera* L.) berries are non-climacteric fruits; they have low respiration rates and display high tolerance to postharvest cold stress^2^. Commercial storage of table grapes is carried out at 0 °C in the presence of SO_2_-emitting sheets to effectively inhibit the development of fungi, especially *Botrytis cinerea*^3^. CA and MA are widely used in postharvest storage of various produce^4^ but in table grapes, these technologies have been explored only in recent years, as alternatives to prevailing practices^5^.

Although it is assumed that changes in table grape composition during cold storage are minimal, little is known about the metabolic processes that occur in grapes during postharvest cold storage. There is evidence that short-term exposure of table grapes to high CO_2_ levels (20 kPa) for 48 to 72 h affects antioxidant activity, *trans*-resveratrol levels in berry skin and expression of genes associated with the phenylpropanoid pathway^6,7^. Prolonged or suboptimal applications of CA or MA may cause a shift to alcoholic fermentation and development of off-flavors, because of accumulation of volatile pyruvate-degradation products, such as ethanol, acetaldehyde, ethyl acetate and acetone^8^. Although off-flavors have been reported to develop following CA storage of grapes^9^, information is lacking on the types and levels of the specific metabolites involved.

The objective of the present research was to examine the effects of long-term postharvest treatments on flavor and health-promoting properties of table grapes. The results demonstrate that cold storage induced the accumulation of specific compounds with recognized health-promoting properties in grapes, whereas storage under reduced O_2_ and elevated CO_2_ levels impaired this effect. We also identified a set of compounds and metabolic processes associated with the development of off-flavors under limited O_2_ and elevated CO_2_ levels. This research may, therefore, open the way to understanding how storage conditions modulate gene expression; from a practical standpoint, it may lead to better tools for monitoring postharvest storage of table grapes.

## Results

### Off-flavors developed under elevated CO_2_ levels are associated with the accumulation of volatile compounds related to glycolysis

**Figure 1:**
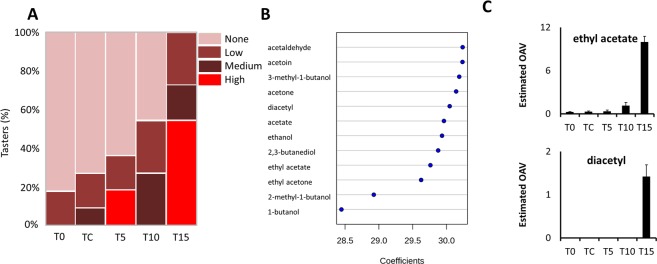
Increased levels of volatile compounds under elevated CO_2_ conditions are associated to off-flavor in table grapes. Grape berries of ‘Superior Seedless’ after harvest (T0), after storage for 6 weeks at low temperature (TC) or after storage at low temperature under constant O_2_ level of 5 kPa and CO_2_ levels of 5, 10 and 15 kPa (T5, T10 and T15, respectively). (**A**) Mosaic plot based on sensory analysis results of T0, TC, T5, T10 and T15. Data shows percent of tasters that identified the berries as having no, low, medium or high off-flavor (**B**) PLS-DA coefficient scores of volatile compounds associated to high off-flavor in T0, TC, T5, T10 and T15 (Supplementary Fig. 1) and (**C**) estimated odor active value (OAV) of two compounds associated with off-flavor after harvest or after storage under different conditions (Supplementary Table 2). Levels of metabolites were analyzed based on three biological replications for each treatment (n = 3).

Clusters of ‘Superior Seedless’ grapes were sampled after harvest (T0), after storage at low temperature for 6 weeks (TC) or after storage at low temperature under a constant O_2_ level of 5 kPa and CO_2_ levels of 5, 10 or 15 kPa (T5, T10 and T15, respectively). Figure 1A shows the percentages of tasters that categorized the grape berries as having no off-flavor or three levels of off-flavor. Half of the tasters noticed low or medium off-flavor at 10 kPa CO_2_; all tasters noticed off-flavor at 15 kPa CO_2_, half of them registering high off-flavor scores. Partial least square - discriminant analysis (PLS-DA) for correlation between volatile compounds and off-flavor levels showed separation into three clusters: T0 first; TC, T5 and T10 joint second; and T15 third (Supplementary Fig. S1A). Component 1 (67.6%) separated T15 from the other four. Therefore, correlation coefficients were examined for volatile compounds that correlated with T15 (high off-flavors) more strongly than with T0, TC, T5 and T10 (Supplementary Fig. S1B). Twelve volatile compounds that were associated with perceived off-flavors included: pyruvate-degradation metabolites such as ethyl acetate, acetone, acetaldehyde, acetate, acetoin; and volatile compounds derived from diacetyl or branch-chain amino acids, such as 3*-*methyl*-*1*-*butanol (L-leucine) (Fig. 1B, Supplementary Table S1).

Odor active values (OAV) serve as an estimator for the potential of a metabolite to affect the flavor of a given sample; values greater than one indicate a higher potential and represent higher concentrations of a given compound than its known odor threshold. Ethyl acetate had an OAV of 10 and diacetyl one of 1.4 in T15 (Fig. 1C); thus they are likely to emit disagreeable odors reminiscent of nail polish or milky compounds, respectively (Supplementary Table S2).

### A shift towards up-regulation of genes under elevated CO_2_ and down-regulation of genes under cold stress

To further explore mechanisms underlying off-flavor accumulation, RNAseq analysis was applied to the grapes under T0, TC, and T15 conditions. Heat-map analysis revealed the reproducibility of the replicates and the significant differences among the treatments (Supplementary Fig. S2): a total of 6600 genes changed under the three sets of experimental conditions and the two thresholds used — LogFC > 1.5 and FDR < 0.05). Under cold-stress conditions 359 genes were up-regulated and 550 down-regulated with respect to both TC and T0; conversely, under anaerobic stress 522 genes were up-regulated and 168 down-regulated with respect to both TC and T0 (Supplementary Fig. S3, Supplementary Tables S3 and S4). Gene ontology analysis that compared the up-regulation of the molecular function of genes under T15 conditions with that under either TC or T0 showed greater overall up-regulation of oxido-reductase activity, of transcription factors and of sequence-specific DNA binding elements (Supplementary Fig. S4). Interestingly, in addition to the effect of the elevated CO_2_ treatment on berry flavor and metabolism, global pathways enrichment analysis showed induction of the phenylpropanoid pathway for both in TC and T15 as compared to T0, suggesting that it is a cold-stress-related effect (Supplementary Tables S5–S14).

### Effects of elevated CO_2_ level on genes involved in off-flavor-related metabolic processes

**Figure 2:**
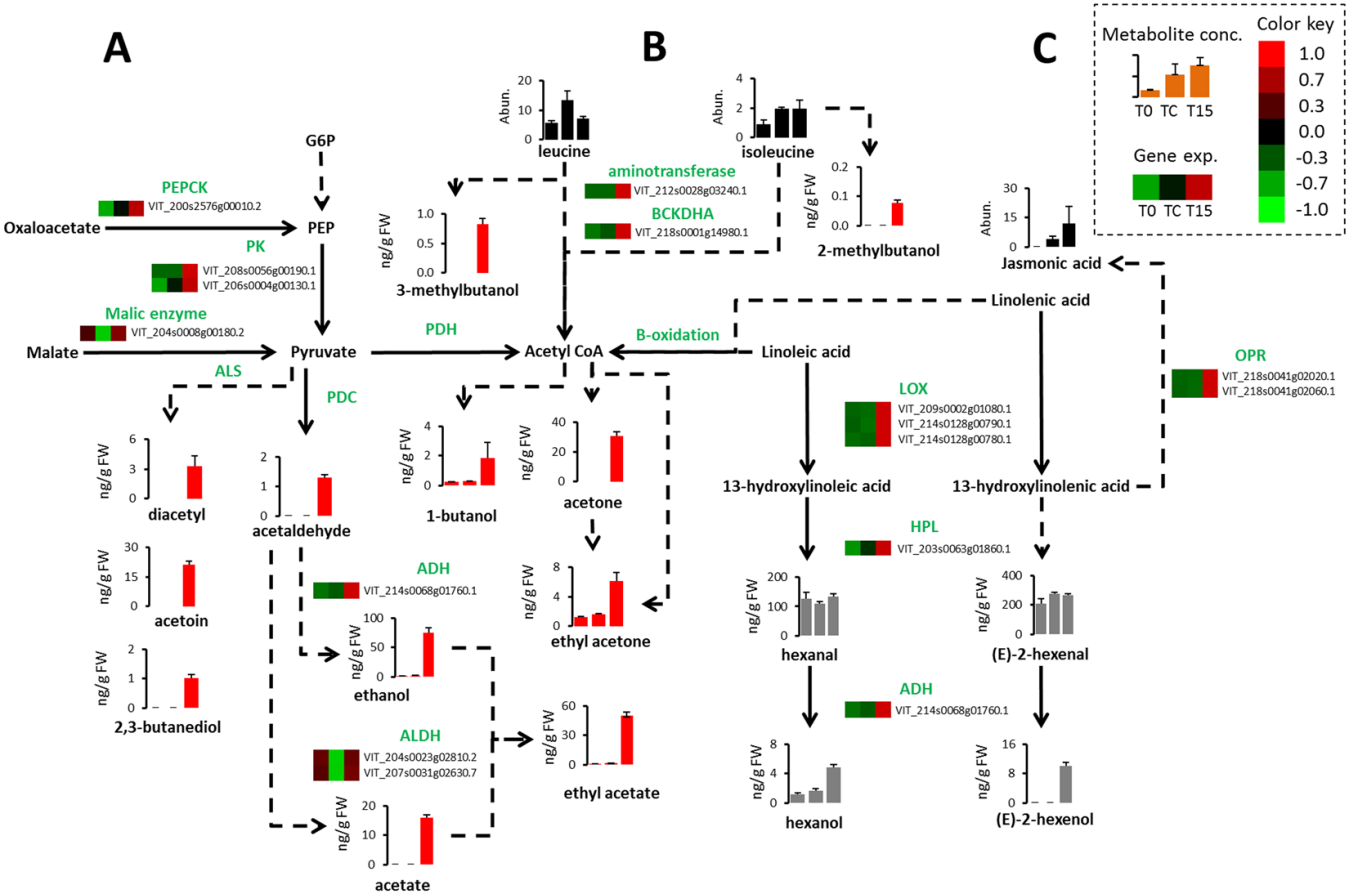
Upregulation of genes putatively involved in metabolism of pyruvate, amino and fatty acid may result in accumulation of volatile compounds associated to off-flavor. Volatile compounds derived from degradation of (**A**) pyruvate, (**B**) branch-chain amino acids or (**C**) fatty acid degradation were examined after harvest (T0), after storage for 6 weeks at low temperature (TC) or after storage at low temperature under O_2_ level of 5 kPa and CO_2_ levels of 15 kPa (T15). Genes are presented in the heat-map analysis if in at least one of the treatments (T0, Tc or T15), genes were down- or upregulated with LogFC > (1.5) and FDR < 0.05. Heat-map was performed based on normalized read counts for the significantly regulated genes: red box indicates upregulated and green downregulated expression. In the heat-maps and in the bar graphs, samples are arranged, from left to right, as T0, TC and T15. Metabolites in graph bars marked in red are associated to off-flavor while grey bars are not associated to off-flavor. *PEPCK*, *phosphoenolpyruvate carboxykinase*; *PK*, *pyruvate kinase*; *PDH*, *pyruvate dehydrogenase*; *PDC*, *pyruvate decarboxylase*; *ALDH*, *aldehyde dehydrogenase*; *ADH*, *alcohol dehydrogenase*; *BCKDHA*, *branched-chain alpha-keto acid dehydrogenase*; *LOX*, *lipoxygenase*; *HPL*, *hydroperoxide lyase*; *OPR*, *12*-*oxophytodienoate reductase*. Dashed lines represent multi-step or unknown metabolic process while full line represents a single known enzymatic step. Units for volatile metabolites are in ng/g FW while values for leucine, isoleucine and jasmonic acid are in peak abundance (Abun.). Levels of metabolites and expression of genes were analyzed based on three biological replications for each treatment (n = 3). Statistical analysis of metabolites is presented in Supplementary Tables 1 and 22.

Genes related to pyruvate metabolism that are up-regulated following exposure to high levels of CO_2_ can be hypothesized to be involved in accumulation of off-flavor related metabolites (Fig. 1B). A *pyruvate kinase* (*PK*) ortholog encoding for the last enzyme in pyruvate biosynthesis, in which phosphoenolpyruvate is converted to pyruvate, was up-regulated in T15 as compared to either TC or T0 (Fig. 2, Supplementary Table S15), as also were two additional genes that may be of importance for accumulation of pyruvate. *Phosphoenolpyruvate carboxykinase* (*PEPCK*), encoding for an enzyme responsible for conversion of oxaloacetate to phosphoenolpyruvate; and an ortholog of *NADP-dependent malic enzyme* gene that encodes for an enzyme that catabolizes malate to pyruvate were both significantly up-regulated in T15 than in TC.

Three gene families are normally involved in conversion of pyruvate to volatile metabolites: *pyruvate decarboxylase* (*PDC*) converts pyruvate to acetaldehyde, *pyruvate dehydrogenase* (*PDH*) converts pyruvate to acetyl-CoA, and *acetolactate synthase* (*ALS*) converts pyruvate to acetolactate. Interestingly, and despite the increase in the metabolites produced by these enzymes, no up-regulation was observed for *PDH*, *PDC* or *ALS* in any of the three treatments, and all gene families had members that were constitutively expressed (Supplementary Table S15).

Downstream along the pathway, two gene families are involved in the metabolism of aldehydes: *alcohol dehydrogenase* (*ADH*) and *aldehyde dehydrogenase* (*ALDH*). A grapevine *ADH* ortholog with multiple isoforms had higher expression in T15 than in T0 or TC (Supplementary Table S15). The *ALDH* family, which encodes for an enzyme that catalyzes the conversion of acetaldehyde to acetate, had two gene members with significantly greater up-regulation in T15 than in TC but not greater than in T0.

Acetyl Coenzyme A (Ac-CoA) is a key metabolic intermediate that may lead to production of acetone and ethyl acetone (2-pentanone). Alternative pathways to the production of Ac-CoA by *PDH* are degradation of branch-chain amino acids, and β-oxidation of fatty acids. Two putative genes involved in the formation of Ac-CoA from leucine and isoleucine were up-regulated: aminotransferase and *branched-chain alpha-keto acid dehydrogenase* (*BCKDHA)* (Fig. 2, Supplementary Table S15). With regard to degradation of linoleic and linolenic acids, several members of the *LOX*, *HPL* and *ADH* gene families were up-regulated in T15 as compared to T0 or TC (Fig. 2, Supplementary Table S15); they encode for enzymes involved in the production of C_6_ alcohols. Although hexanol and (*E*)-2-hexenol increased in T15, they were less likely to contribute to off-flavor in grape berries. Interestingly, jasmonate, another product of this pathway was also found at a higher level in T15 than in T0 or TC, concomitantly with the up-regulation of putative *12-oxo-phytodienoic acid reductase* (*OPR*) gene, which is known to be involved in jasmonate biosynthesis (Fig. 2, Supplementary Table S15).

### Major shifts in regulation of gene expression under elevated CO_2_ levels and low temperature

**Figure 3:**
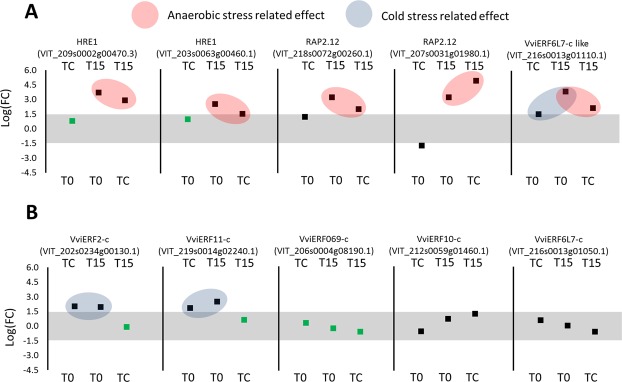
Suspected hypoxia-response elements (*HRE*) upregulated under elevated CO_2_ levels. Grape berries of ‘Superior Seedless’ after harvest (T0), after storage for 6 weeks at low temperature (TC) or after storage at low temperature under O_2_ level of 5 kPa and CO_2_ levels of 15 kPa (T15). The Y-axis refer to LogFC values. Comparison is between pairs of treatments at the top and bottom of each panel. Grey surface between LogFC < (1.5) represent comparison that did not change significantly. Red balloons represent conditions of genes that were upregulated in T15 as compared to T0 and TC and therefore are related to anaerobic stress. Blue balloons represent conditions of genes that were upregulated in TC and in T15 as compared to T0, but not between T15 to TC and therefore are related to cold stress. Squares indicate the mean Log(FC): black squares indicate FDR < 0.05; green squares indicate FDR > 0.05. Expression of genes were analyzed based on three biological replications for each treatment (n = 3).

Expression of 412 putative and characterized transcription factors was examined among the three treatments (Supplementary Tables S16–S19). The ethylene response factors (*ERF*s) consist of a subfamily termed “*Hypoxia-Response Elements*” (*HRE*s), which are active under anaerobic stress. Analysis of *ERF*s related to anaerobic stress was focused on the three members of the *HRE* family characterized in *Arabidopsis*^10^: *HRE1* (At1g72360), *HRE2* (At2g47520), and *RAP2.12* (*At1g5*3*910*). Four putative *ERF*s, two of which are similar to *AtRARP2*.12 and two to *HRE1* were significantly up-regulated in T15 compared to TC or T0 (Fig. 3, Supplementary Table S16). Another five *ERFs* were reported elsewhere to be induced following 3 days of exposure to high CO_2_ in *V. vinifera* ‘Cardinal’ berries^11^ but none of them was up-regulated in T15 as compared to TC or T0. A gene with high similarity to one of the five known *ERFs* from *V. vinifera* — *VvERF6L7-c-like gene* — displayed significantly higher expression in T15 and in TC than in T0 (Supplementary Table S16). The *Arabidopsis* hemoglobin-like gene *AtHB1* (NP_179204.1 was up-regulated under anaerobic conditions. Among the several *V. vinifera* orthologous *HB1* genes, one gene — VIT_203s0063g01960.1 — was up-regulated in T15 as compared to TC or T0 (Supplementary Table S20). *C-repeat binding factors* (*CBF*s), transcription factors^12^, and their inducers (*ICE*s)^13^ were shown to be involved in cold-stress response and freezing tolerance in *V. vinifera*. However, we did not observe increased expression of any of the *VvCBFs* in T15 or TC as compared to T0. *VvICE1*, an inducer of *CBF* expression was up-regulated in T15 and TC as compared to T0 (Supplementary Table S19). The CBFs are also called dehydration responsive element-binding (DREB)^14^. Out of the 38 partially characterized *VvDREB*s, four were significantly changed: *VvDREB05* and *VvDREB08* were down-regulated by cold stress, *VvDREB12* also was down-regulated under cold stress but with stronger preference for anaerobic conditions, whereas *VvDREB25* was up-regulated in T15 as compared to TC but not as compared to T0 (Supplementary Table S19). *VvDHN1a*, which is a structural gene encoding dehydrin 1a that is associated with the cold-stress response was up-regulated in TC and T15 as compared to T0 (Supplementary Table S21).

### Metabolic changes in phenylpropanoid levels under low temperature and elevated CO_2_ levels

**Figure 4:**
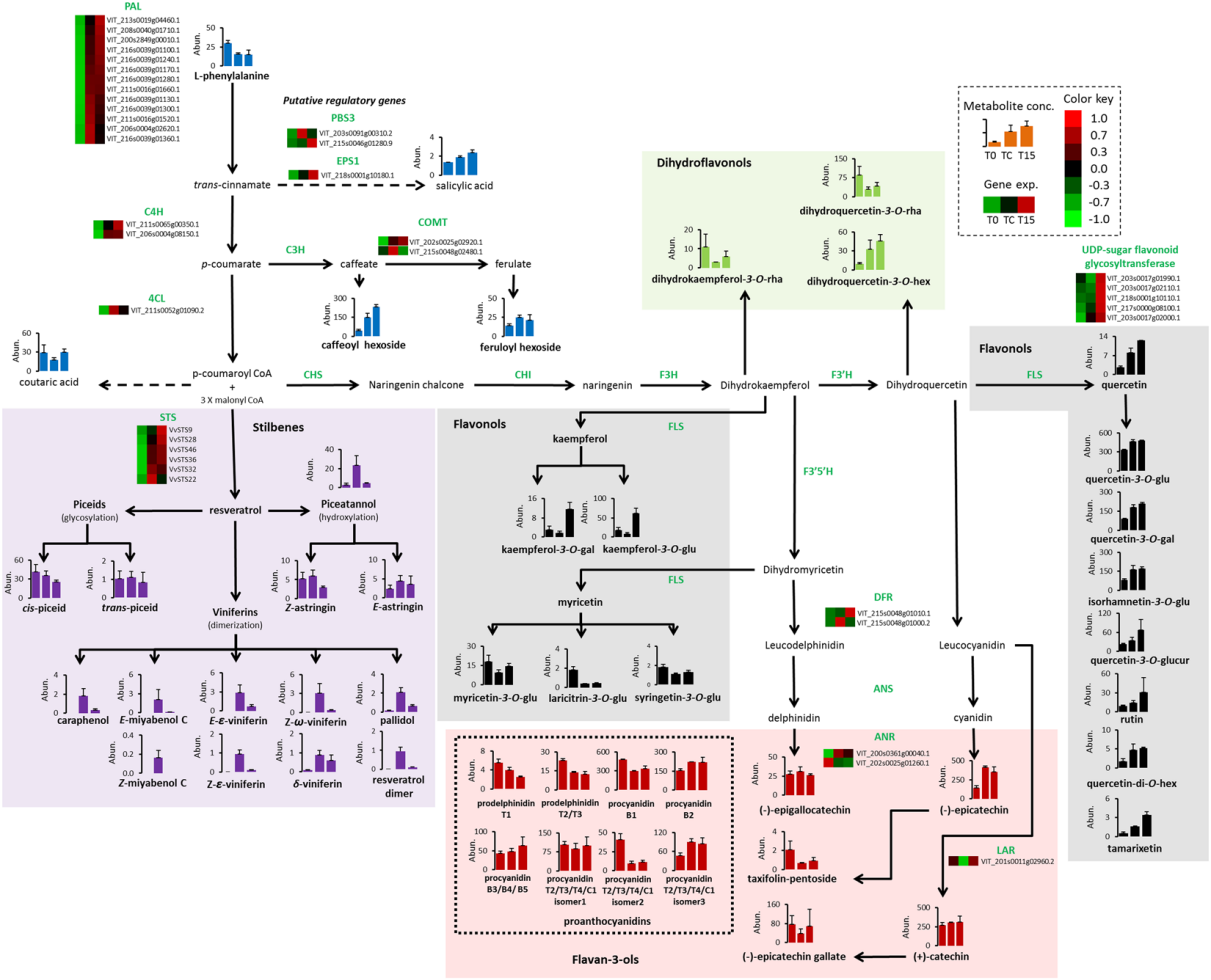
Upregulation of the phenylpropanoid pathway resulted in the accumulation of metabolites from different sub-branches under cold stress and elevated CO_2_ levels. Grape berries of ‘Superior Seedless’ after harvest (T0), after storage for 6 weeks at low temperature (TC) or after storage at low temperature under O_2_ level of 5 kPa and CO_2_ levels of 15 kPa (T15). Graphs are colored according to the following key: flavonols (black), dihydroflavonols (green), flavan-3-ols and their derivative proanthocyanidins (red), stilbenoids (purple). Gene are presented in the heat-map analysis if in at least one of the treatments (T0, Tc or T15), genes were down- or upregulated with LogFC > (1.5) and FDR < 0.05. Heat-map was performed based on normalized read counts for the significantly regulated genes: red boxes indicate upregulated and green downregulated expression. Bar graphs describe changes in metabolite accumulation (Y-axis: relative abundance). In the heat-maps and in the bar graphs, samples are arranged, from left to right, as T0, TC and T15. *PAL*, *phenylalanine ammonia lyase*; *C4H*, *cinnamate 4-hydroxylase*; *4CL*, *4-coumarate-CoA ligase*; *STS*, *stilbene synthase*; *CHS*, *chalcone synthase*; *CHI*, *chalcone isomerase*; *F3H*, *flavanone 3-hydroxylase*; *F3*′*H*, *flavonoid 3*′*-hydroxylase*; *FLS*, *flavonol synthase*; *F3*′*5*′*H*, *flavonoid 3*′,*5*′*-hydroxylase*; *DFR*, *dihydroflavonol 4-reductase*; *ANS*, *anthocyanidin synthase*; *ANR*, *anthocyanidin reductase*; *LAR*, *leucoanthocyanidin reductase*. *PBS3* and *EPS1* two putative regulatory genes those are associated to salicylate biosynthesis. Dashed lines represent multi-step or unknown metabolic process while full line represents a single known enzymatic step. Levels of metabolites and expression of genes were analyzed based on three biological replications for each treatment (n = 3). Statistical analysis is presented in Supplementary Table 22. Units for metabolite levels are in peak abundance (Abun.).

Metabolites of four branches of the phenylpropanoid pathway changed among the treatments, including 14 stilbenes, 13 flavonols, three dihydroflavonols, five flavan-3-ols, and eight of their derivative proanthocyanidins. Of the 47 metabolites detected, 30 had higher level in TC and T15 than in T0 (Fig. 4, Supplementary Table S22). The levels of 22 metabolites were higher in either TC, T15, or both than in T0. The metabolites were detected mainly in their glycosylated forms, such as kaempferol-3-O-galactoside. The levels of the amino acid L-phenylalanine, the entry-point metabolite of the phenylpropanoid pathway, was significantly lower in both TC and T15 than in T0 (Fig. 4, Supplementary Table S22).

### Accumulation of stilbenes under cold stress

One of the important junctions in the phenylpropanoid pathway is the conversion of *p*-coumaroyl-CoA and three molecules of malonyl-CoA to either the stilbene *trans*-resveratrol, or the flavonol naringenin chalcone. The stilbenes detected in the present study included piceatannol, a hydroxylated form of resveratrol, piceids that are glycosylated forms of resveratrol and viniferins that are oligostilbenes.

Only two viniferins — palidol and *δ*-viniferin — were detected in T0. Six viniferins — (*E*)-miyabenol C, (*E*)- and (*Z*)-*ε*-viniferin, caraphenol, (*Z*)-*ω*-viniferin and an unidentified resveratrol dimer — were detected in TC and T15, and (*Z*)-miyabenol C was detected only in TC (Fig. 4, Supplementary Table S22). Of the eight resveratrol oligomers present, seven were more abundant in TC than in T0 or T15; Only *δ*-viniferin was found at similar levels in TC and T15, both higher than in T0 (Fig. 4, Supplementary Table S22).

The two glycosides of piceatannol *(E)-* and (*Z*)*-*astringin, did not show any significant difference between the treatments. No significant changes were detected for *cis*- or *trans*-piceid isomers among T0, TC and T15 (Fig. 4, Supplementary Table S22). Interestingly, in all treatments, *cis*-piceid was 40-fold more abundant than the *trans* isomer. BLAST of *hsaCYP1B1* (Genbank NP_000095.2), a *cytochrome P450* (*CYP*) gene encoding *trans*-resveratrol hydroxylase, identified a predicted *CYP* that was upregulated in TC compared to T15 (Supplementary Table S23).

### Accumulation of flavonoids under elevated CO_2_ levels

The flavonol subgroup was represented by 13 metabolites in ‘Superior Seedless’ grape berries. Two kaempferol-derived compounds — kaempferol-*3*-*O*-galactoside and kaempferol-3-*O*-glucoside — were more abundant in T15 than in T0 or TC, as also were quercetin and tamarixetin. Additional quercetin derivatives, such as quercetin-3-*O*-galactoside and quercetin-3-*O*-glucoside, were more abundant in T15 and TC than in T0. The three flavonols derived from myricetin showed the opposite trend: laricitrin-3-*O*-glucoside content was higher in T0 than in TC or T15, and myricetin-3-*O*-glucoside and syringetin-3-*O*-glucoside were more abundant in T0 than in TC, but not more than in T15 (Fig. 4, Supplementary Table S22).

Dihydroflavonols did not show a consistent pattern of accumulation among treatments: dihydrokaempferol-3*-O*-rhamnoside levels did not differ significantly among the treatments; the dihydroquercetin-3-*O*-rhamnoside level was higher in T0; and dihydroquercetin-3*-O*-hexoside was more abundant in TC and T15 than in T0.

Thirteen flavan-3-ols and their polymerized derivatives — proanthocyanidins — were detected in the berries. We identified four flavan-3-ols: (−)-epicatechin, (−)-epigallocatechin, (+)-catechin, and (−)-epicatechin gallate, but only (−)-epicatechin was affected by the treatments; it was more abundant in T15 and TC than in T0 while taxifolin-pentoside showed the opposite trend. Proanthocyanidins displayed a different pattern from that observed for stilbenes or flavonols: isomer 1 of procyanidin T2/T3/T4/C1 was not affected by the treatments, whereas isomer 2 accumulation was higher in T0 than in TC or T15, and isomer 3 accumulation was higher in TC and T15 than in T0 (Fig. 4, Supplementary Table S22).

### Induction of phenylpropanoid pathway genes under low temperature and elevated CO_2_ levels

Analysis of the expression levels of genes encoding for the 16 enzymatic steps of the phenylpropanoid pathway demonstrated that 7 steps displayed significant changes in T15 or TC compared to T0 (Fig. 4, Supplementary Table S24). The initial reaction of the phenylpropanoid pathway involves three key steps: *phenylalanine ammonia lyase* (*PAL*), *cinnamate-4-hydroxylase* (*C4H*) and *4-coumarate-CoA ligase* (*4CL*) with, respectively, 13, 2 and 1 gene(s) that were significantly up-regulated in TC and T15 as compared to T0. Downstream, the pathway splits into two branches, stilbenes and flavonols, that are catalyzed by *stilbene synthase* (*STS*) and *chalcone synthase* (*CHS*) gene products, respectively. Both *STS* and *CHS* encode for enzymes that utilize the same substrates — *p*-coumaroyl-CoA and three molecules of malonyl-CoA — to produce resveratrol or naringenin chalcone, respectively, and the genes share many features^15^. *STS* had six homologous genes that were significantly up-regulated in TC and T15 as compared to T0, whereas no up-regulation was observed for *CHS* (Fig. 4). Peroxidase activity was associated with the biosynthesis of viniferins^16^. Of the 73 putative genes encoding for peroxidases (EC 1.11.1.7), only two were up-regulated in TC as compared to T0 or T15 (Supplementary Table S25). Peroxidase 16 (VIT_211s0052g00650.1) had logFC of 3.14 as compared with T0, and logFC of 4.07 as compared with T15.

Downstream in the flavonoid branch there were significant changes in expression of members of *dihydroflavonol 4-reductase* (*DFR*)*, anthocyanidin reductase* (*ANR*) and *leucoanthocyanidin reductase* (*LAR*) among the treatments but without conclusive correlation to the respective metabolite levels. Five putative *flavonoid glycosyltransferase* orthologues encoding enzymes that add sugar moieties to flavonoids were upregulated in T15 compared to TC and T0 (Fig. 4, Supplementary Table S24).

### Regulation of the phenylpropanoid pathway

**Figure 5:**
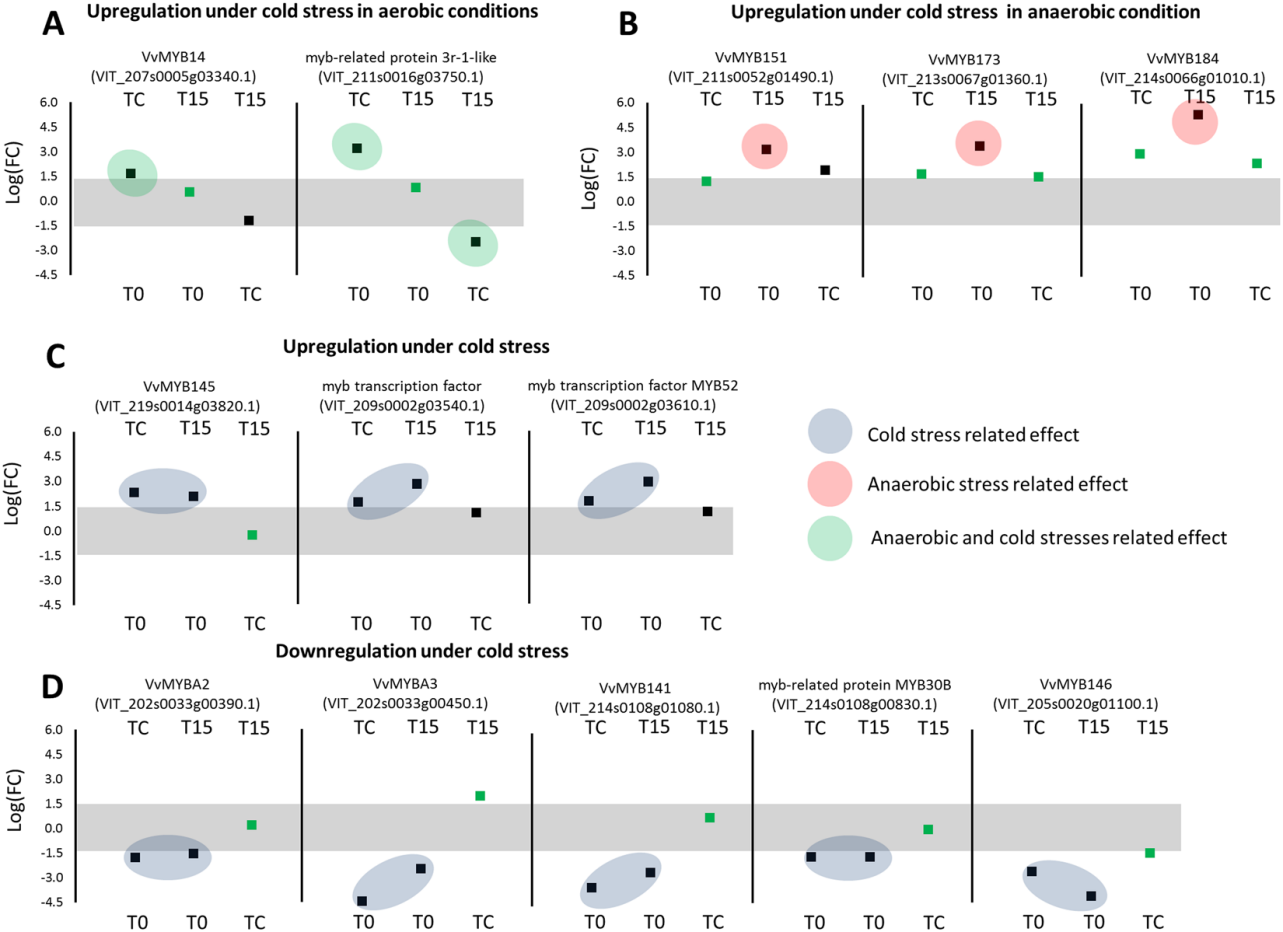
*MYB* transcription factors significantly affected by cold stress and elevated CO_2_ levels. Results appear as comparisons between two treatments. Grape berries of ‘Superior Seedless’ after harvest (T0), after storage for 6 weeks at low temperature (TC) or after storage at low temperature under O_2_ level of 5 kPa and CO_2_ levels of 15 kPa (T15). Comparison is between pairs of treatments at the top and bottom of each panel. The Y-axis refer to LogFC values. Grey surface between LogFC < (1.5) represent comparison that did not change significantly. Red balloons represent upregulation of genes at T15 as compared to T0 and TC (anaerobic stress). Blue balloons represent upregulation of genes at TC and T15 as compared to T0, but not between T15 to TC (cold stress). Green balloons represent upregulation of genes at TC as compared to T0 or T15 (cold stress under aerobic conditions). Squares indicate the mean Log(FC): black squares indicate FDR < 0.05; green squares indicate FDR > 0.05. Expression of genes were analyzed based on three biological replications for each treatment (n = 3).

*MYB* transcription factors are known to play key roles in regulating the phenylpropanoid pathway^17–20^. Recent studies^21,22^ extended the list of *MYB*s and *WRKY*s that are presumably involved in stilbene biosynthesis, and improved their annotation; 21 *MYBs* showed significant up- or down-regulation under the experimental setup (Supplementary Table S18). Two *MYBs* were up-regulated in TC as compared to T15 or T0: the stilbene regulator *VvMYB14*^20^, and MYB-related protein 3r-1-like (Fig. 5A, Supplementary Table S18). *VvMYB15*, another stilbene regulator^20^ was up-regulated in T15 as compared to T0 but not with respect to TC (Supplementary Table S18). Three *MYB*s were up-regulated under cold and anaerobic conditions (Fig. 5B, Supplementary Table S18) and another three *MYBs* were up-regulated under cold stress without association with higher CO_2_ levels (Fig. 5C, Supplementary Table S18). Conversely, five *MYBs* were down-regulated under cold-stress conditions (Fig. 5D, Supplementary Table S18). The family of WRKY transcription factors was also implied to regulate the phenylpropanoid pathway^22^ and 20 WRKYs changed in T15 or TC as compared to T0. *VvWRKY24* was highly up-regulated under cold stress whereas *VvWRKY44* underwent significant changes in expression among the treatments (Supplementary Tables S17). *PBS3* and *EPS1* are two regulators of the salicylate pathway and putative homologs of these genes were up-regulated in T15 and TC than in T0 (Fig. 4, Supplementary Table S26).

## Discussion

Berry flavor was examined in five storage treatments, including low temperature and elevated CO_2_ levels. The treatment with the highest CO_2_ level of 15 kPa (and 5 kPa O_2_) resulted in a significant negative effect on berry flavor during 6 weeks of storage. The 12 metabolites that increased in T15 (elevated CO_2_ levels, i.e., anaerobic conditions) were derived mainly from pyruvate degradation with two representatives from branch-chain amino acids. Of the 12 compounds that had increased levels during storage under elevated CO_2_ levels, two volatile compounds — diacetyl and ethyl acetate — showed OAV scores >1, a threshold value that indicates the potential to contribute to grape flavor. Ethyl acetate has been associated with off-flavors in fruits, including strawberries and mandarins^23,24^ but not in grapes. Diacetyl was associated with PDC activity^25,26^ in various fruits and was reported as a potential metabolite that contributed to off-flavors as a result of microbial activity^27,28^. Although the contributions of individual volatile compounds to off-flavor are well established, the combined effect of the mixture cannot be ignored^29^. For example, diacetyl and acetoin share a similar ‘milky’ odor character that could be enhanced by the presence of both compounds in the medium and under elevated ethanol levels (i.e., in berry fermentation). Our present results associate the accumulation of suspected off-flavor volatile compounds in grapes with up-regulation of genes such as *PK*, *PEPPCK*, and *malic enzyme*, from various upstream branches involved in the production of pyruvate. These results indicate that increased pyruvate flux is a more likely lead to up-regulation of pyruvate degradation, because no up-regulation was recorded for *PDC*, *PDH* or *ALS*.

The sub-branch of pyruvate degradation by the *ALS* gene family encoding enzymes that convert pyruvate to acetolactate has only one member characterized in *Arabidopsis*^30^. The *ALS* activity is followed by a still unknown enzymatic activity or by a spontaneous step that leads to accumulation of diacetyl, its conversion to acetoin and, in turn, to the conversion of acetoin to 2,3-butanedienol by dehydrogenases that have not so far been identified in plants. Acetoin, diacetyl, and 2,3-butanedienol increased in various wine grape cultivars undergoing carbonic maceration, but their accumulation patterns varied substantially among varieties and the accumulation was attributed to microbial rather than endogenous activity^31^. One of the largest families of genes in plants, the *ADH*s, also is associated with off-flavors in numerous fruits; ADH enzyme activity was associated with off-flavors in mandarins, grapefruits^32^ and strawberries^33^. Moreover, *ADH*s genes and their isoforms have varied substrate specificity^34^; in grapes six *VvADHs* were characterized either by sequence identity^35^ or by functional expression^36^. Moreover, VvADH enzyme activity was shown to be affected by the hypoxic conditions in grape cell culture^37^; in contrast to our present experimental conditions, stress duration was relatively short and ambient temperature (20 °C) was used. Except for the six characterized *VvADHs* genes, additional putative members of the ADH family were found by sequence identity but their functions or activities in grape berries were not described^35^. Grapevine leaves showed higher levels of glycosylated volatile alcohols from various metabolic pathways, e.g., monoterpenols or C_6_ alcohols in transgenic grapevine overexpressing *VvADH2*^38^. So far a major part of the study of *VvADH* in grapes was focused on grapevine and berry development^39^. Our present results suggest that members of the *VvADH* gene family that are constitutively expressed or, possibly, that up-regulation of a specific *VvADH* — VIT_214s0068g01760.1 and its isoforms — might be involved in the production of multiple volatile compounds under anaerobic conditions (T15) that can associated with off-flavors; such compounds include: ethanol, acetaldehyde, ethyl acetate, 2-methylbutanol and 3-methylbutanol^23,24,31^. Conversely, ADH enzyme also involved in the last step of biosynthesis of hexanol or (*E*)-2-hexenal, which are not associated with off-flavor in table grapes^40^. Except for the *ADH* gene family, the *LOX*-*HPL* genes were also shown to be important for the diversification of C_6_ volatiles in the berries^41^. Characterization of the *LOX* gene family in *Vitis vinifera* identified 18 putative genes that were partially characterized for 13-LOX activity^42^. The *HPL* gene family is part of the *cytochrome P450*s superfamily and may hold a substrate specificity to either 13-HPL or 9-HPL or to both; so far, two HPLs were characterized in *Vitis vinifera*^43^. The up-regulation of expression of *ADH* and *HPL* genes is consistent with the increase in the C_6_ volatile, and up-regulation of the *LOX* can be associated with the increase in jasmonate under anaerobic conditions.

The increased expression of genes involved in fermentative respiration under anaerobic conditions is mediated by specific transcription factors, some of which have been identified in model systems and in table grapes^10,44,45^. A unique and characterized group of *ERFs* — *HRE*s^10^ — was shown to be involved in the transition to fermentative respiration in *Arabidopsis*. *HRE* genes belong to the group of VII *ERF*s and respond to low-oxygen conditions^10^. The superfamily of *ERF* genes was characterized in grapevine and was shown by sequence identity to include over 100 genes^46,47^. The HRE2 protein is stabilized under hypoxic conditions by a conserved motif that starts with Met-Cys in its N-terminus^48,49^.

Under our experimental conditions, the expression of five grape orthologs to *HRE*s increased under elevated CO_2_ conditions, making them also plausible candidates for regulation of hypoxia responses. Conversely, five *VvERF*s that were previously shown to be up-regulated in grape berries under elevated CO_2_ levels, were not up-regulated under T15 as compared to TC and genes (Fig. 3). *V viERF2-c* and *VviERF11-c*, were upregulated following cold stress conditions (upregulation in TC and in T15 as compared to T0) . One of the major differences between our present conditions and that work^11^ was the time factor: the grape berries were exposed to the elevated CO_2_ for only 3 days as compared to 6 weeks in our experiment. Six *ERF*s in addition to the five *HRE* orthologous genes were induced under our long-term exposure to elevated CO_2_ conditions; their putative functions and responsiveness to anaerobic stress should be evaluated under different conditions^50,51^. Up-regulation of *ADH* and *HB1* (*hemoglobin*) was previously suggested as a hallmark of anaerobic conditions^10^ and our present findings support this notion for *HB1* and one of the *VvADH* family members. *AtHB1* encodes a non-symbiotic hemoglobin-like protein that increases in *Arabidopsis* under anaerobic conditions. The non-symbiotic hemoglobin class 1 proteins are up-regulated under hypoxic conditions and possess high affinity to O_2_, whereas hemoglobin class 2 proteins possess low affinity to O_2_ and are up-regulated under cold stress^52^. *HB1* was hypothesized to act as a plant sensor for hypoxic conditions^53^ and it may be an important marker also for the development of anaerobic stress during cold storage of grapes.

Transcription factors that were shown to be up-regulated under cold stress included several *ERF* gene family members, *MYB*s and *WRKY*s that were shown to be up-regulated after short exposure to cold stress^49,54^. We did not detect up-regulation of any of the previously characterized *VvCBF* genes that previously were associated with cold stress^55,56^; in contrast, the putative *CBF* inducer, *VvICE1*, was up-regulated under cold stress. In grapes, the *VvCBF*s and *VvICE*s were examined for their effects on plant growth but their expression was not examined at the fruit level^55,56^; future studies should examine the response of *VvCBFs* to short- and long-term low temperature at the berry level. It should also be pointed out that *VvCBF1* (AY390372) was not identified in the *V. vinifera* genome and that functional analysis of the *VvCBF*s and *VvICE*s is desirable. Apart from the relatively well characterized function of *DREB1-4*^57–59^, there has been no good characterization of additional gene family members. Two *VvDREB* genes — *VvDREB05* and *VvDREB08 —* that showed down-regulation under cold stress behaved in accordance with short-term down-regulation^14^ but their role in the cold-resistance mechanism is not well understood.

The phenylpropanoid pathway is responsible for the synthesis of important compounds involved in various stress responses in plants^60^, including acclimation to cold stress^61^; in grape berries, this pathway has a central role in the biosynthesis of compounds that impart important quality characteristics, such as antimicrobial activity, color, flavor, antioxidant capacity and astringency. Reduced levels of L-phenylalanine in treatments TC and T15, along with up-regulation of *PAL*, suggest that more L-phenylalanine entered the pathway and subsequently was metabolized to downstream metabolites. This finding is consistent with the increased levels of most phenylpropanoids detected in the berries indicating on the importance of inducing the initial stage of the phenylpropanoid pathway to produce various metabolites downstream along the pathway and its branches. Previous studies demonstrated specific metabolic and transcriptomic changes in the phenylpropanoid pathway following exposure of ‘Cardinal’ grape berries to cold stress under aerobic or short-term anaerobic conditions^6,7,11^. In general, our present findings are in agreement with these studies with respect to the up-regulation of *PAL* and *STS*, but offer a perspective on how prolonged cold stress or elevated CO_2_ levels might affect gene expression and accumulation of phenylpropanoid pathway metabolites during storage.

One main branch of the phenylpropanoid pathway is that of the flavonols such as quercetin, kaempferol and myricetin, and their glycoside derivatives, which exhibit antioxidant and anti-inflammatory activities^62^ and were repeatedly reported to be present in grape berries. Our results show that after long exposures to elevated CO_2_ levels, the levels of kaempferol and quercetin glycosides increased significantly. This effect was not demonstrated previously; it may be attributed to a shunting effect that could result from inhibition of stilbene synthesis under elevated CO_2_ levels.

Stilbenes are another important branch of the phenylpropanoid pathway that is involved in the production of resveratrol and its derivatives; resveratrol derivatives with health-promoting properties for humans include viniferins^63,64^, which were shown to strongly suppress proliferation of various cancerous cell lines and to be involved in apoptosis^65,66^. Seven out of the nine viniferins identified in the present study appeared only in TC, and the other two viniferins increased in TC as compared to T15 and T0. *Trans*-resveratrol can be converted to δ-viniferin by horseradish peroxidase enzyme extracts^16^; we detected increases in four peroxidase-like gene homologs concurrently with the accumulation of δ-viniferin, which raised the hypothesis that grape peroxidases might be involved in the biosynthesis of δ-viniferin. The hydroxylated analog of *trans*-resveratrol — piceatannol — was 10 times as abundant in TC as in T0 or T15. The conversion of *trans*-resveratrol to piceatannol occurs in humans *via* the action of *CYP1B1*^67^ but, to the best of our knowledge, neither this enzymatic step nor the gene encoding a putative enzyme catalyzing this reaction was characterized in plants. We identified an orthologous gene of the *hsaCYP1B1* that potentially could be involved in the hydroxylation of *trans*-resveratrol to piceatannol in grape berries. The up-regulation in *STS* does not account for the increased levels of piceatannol and viniferins; piceids did not accumulate to a greater extent in TC than in T0 or T15. Alternatively, increased expression of peroxidases, as demonstrated in the present study, and of hydroxylases might account for the greater accumulation of the stilbenes in TC than in T0 or T15. Previous findings revealed an increase in resveratrol induced by low-temperature stress before harvest^68^ and a decrease after exposure to cold stress^6^. One explanation for these findings is the conversion of *trans*-resveratrol to piceatannol or viniferins during prolonged cold stress in our experiments, or the fact that ‘Cardinal’, a red grape berry cultivar, is not blocked in anthocyanin synthesis.

The regulation of biosynthesis of stilbenes in plants, and especially in grapes, has attracted much attention, with a focus on the role of *MYB* genes, because many of the *MYB*s regulate various branches of the phenylpropanoid pathway^19,69^. Specifically: two *MYBs* were characterized as *STS* gene regulators (*VvMYB14-15*) and a third also was hypothesized to be an *STS* regulator, in light of its co-expression pattern (*VvMYB13*). *MYBS13-15* were shown to be induced by biotic and abiotic stress conditions and the three regulatory genes form a co-expression network with seven *STS* genes^69^. Under our present experimental conditions, we did not find significant expression of *VvMYB13* and the up-regulation of *VvMYB15* at T15 with respect to T0 could not be associated with stilbene accumulation pattern in the berries. Conversely, our findings show that up-regulation of *VvMYB14* following cold stress (TC) was concomitant with the viniferins accumulation pattern. Thus, our results suggest that *VvMYB14* is involved in the regulation of viniferins or piceatannol in grape berries during postharvest storage. Recently, the role of *WRKY* transcription factors in regulation of stilbene biosynthesis was highlighted^22^; of special interest was the possible interaction of *VvWRKY24* with *VvMyb14* at the promoter level. *VvWRKY24* was also shown to interact with the promoter of *VvSTS29*. The Arabidopsis homolog of *VvWRKY24* (*AtWRKY33*) was shown to be involved in salicylate signaling, redox homeostasis, and jasmonate/ethylene-mediated defense responses^70^. Further analysis and characterization are required for the additional 12 *MYB* and 10 *WRKY* genes that showed differential expression between aerobic and anaerobic conditions under cold stress. One of the hypotheses for the induction of stilbenes at low temperature corresponds to their known protective role as stress-induced phytoalexins^68,71^, but they also mitigate cold–induced ROS damage^72^. Conversely high CO_2_ may inhibit accumulation of resveratrol and its derivative(s) by interfering with ion transport and associated redox processes^71,73^.

Salicylate is a plant hormone known to be involved in the response to abiotic-stress conditions^74^. The biosynthesis of salicylate was ascribed to two pathways. The first is from chorisimate to isochorismate and then to salicylate via two steps involving *isochorismate synthase* (*ICS*) and *isochorismate pyruvate lyase* (*IPL*). However, in plants only the *ICS* gene was identified so far in *Arabidopsis*. The second pathway is from trans-cinnamate to salicylate via an as yet unknown number of steps^75^. Two key regulatory genes — *PBS3* and *EPS1 —* were identified as important for biosynthesis of salicylate; under our experimental conditions both were up-regulated in T15 and TC as compared to T0, and in accordance with the accumulation of salicylate. Exogenous application of salicylate resulted in induction of *PAL*, which catalyzed the conversion of L-phenylalanine to trans-cinnamate^76^. Therefore, as suggested by the data in Fig. 4, it is hypothesized that salicylate also may induce *PAL* in grape berries exposed to cold and anaerobic stresses. Interestingly, *VvWRKY3*, which was up-regulated as a result of anaerobic stress under our experimental conditions, also was shown to be up-regulated by salicylate and jasmonate in leaves of *Vitis pseudoreticulata*^77^.

Jasmonate is another plant hormone that can be activated by either biotic or abiotic stress conditions^78^. Jasmonate biosynthesis is initiated in the plastid by *LOX*^79^ and subsequently the intermediate cis-12-oxo-phytodienoic acid is transported to the peroxisome by an *ATP-binding cassette* (*ABC*) *transporter* and undergoes reduction by *OPR* and a β-oxidation process^80^. *OPR* is the third enzyme in the biosynthesis of jasmonate and it was characterized in different plant species, e.g. *Zea mays*^81^, and putative *VvOPRs* also were described^82^. Application of jasmonate treatments to the berries had resulted in increased *LOX* enzyme activity and enhanced levels of several C_6_ volatiles, including the two alcohols hexanol and *(E)*-2-hexenol, in agreement with our present results^83^.

The induction of putative *LOX* and *OPR* genes under anaerobic conditions (Fig. 2, Supplementary Table S15) was consistent with the increase in jasmonate levels, especially under anaerobic conditions. Interestingly, jasmonate was shown to be involved in the induction of various ‘specialized’ metabolites in plants^84^ and in particular *PAL* expression and stilbene accumulation including in grape berries^85^. A possible explanation for the increased levels of both salicylate and jasmonate under elevated CO_2_ levels is the higher flux of the upstream metabolites, phenylalanine and linolenic acid.

In conclusion: postharvest storage research has focused mainly on developing methodologies to control and minimize detrimental changes in fruits and vegetables during storage. During storage fruits and vegetables exhibit active respiration to meet the energy demands of the cells and, most importantly, they can respond to environmental cues. Our findings shed light on the specific metabolic shifts occurring in table grapes under elevated CO_2_ levels and in cold storage. In the present paper we describe some of the specific genes that may be involved in responding to these conditions and regulating the responses; we also describe specific metabolic shifts and the resulting metabolites that accumulate under cold stress or combined cold and anaerobic stresses. The volatile metabolites that accumulate under anaerobic stress may be useful as off-flavor markers that facilitate improved control of alternative storage conditions. Our results indicate that the levels of stilbenes, normally associated with beneficial health-promoting effects, are more than 60 times as high after storage as before storage. Thus, we demonstrated that storage may have beneficial effects in creating new opportunities to actively enhance specific metabolites after harvest.

## Materials and Methods

### Plant material, experimental setup and storage conditions

Table grapes (*Vitis vinifera* L.) cv. ‘Superior Seedless’ were obtained from a commercial vineyard in Lachish, Israel (lat. 31°33′, long. 34°51′). The vines were supported by a Y-shaped trellis with planting spaces of 3 × 1 m; the shoots were trained to eight canes, with 15 buds per cane. Gibberellin was applied at fruit set at a rate of 15 mg/l directly to the grape clusters. Leaf removal was performed at *veraison*.

Grape clusters were randomly distributed into storage boxes, each containing approximately eight clusters with a total weight of approximately 5 kg. The boxes were placed in 400-l storage chambers. The atmosphere composition was controlled by an ICA6000 system (ICA Group, Cardiff, UK). The treatments were as follows: T0 (not stored) – grapes sampled on day of harvest; TC – grapes kept under 20 kPa O_2_ and 0 kPa CO_2_, designed to simulate atmospheric conditions; T5, T10 and T15 – grapes kept at air compositions of 5 kPa O_2_ and 5, 10 and 15 kPa CO_2_, respectively. Storage conditions for all treatments (except T0) were: 0.0 ± 0.5 °C with RH of 90–95%. Storage duration was 6 weeks followed by shelf life at 20 °C for 2 days. Postharvest analysis was performed only on berries that did not show any sign of external or internal decay.

### Berry weight, firmness, and sugar and acid contents

Berry weight was measured by random sampling of 30 berries in each of three replications. Berry firmness and diameter were measured on 20 berries from each replication with a Firmtech II small fruit firmness analyzer (BioWorks, Wamego, KS, USA). Juice for measurement of total soluble solids (TSS) and TA (titratable acidity) was prepared by maceration of the berries in a T398 juice blender (Grandix, Cheres, France) and filtration through four layers of cheesecloth. The TSS (° Brix) and TA (%), respectively, were determined with a digital refractometer (Atago, Tokyo, Japan) and an autotitrator (Metrohm, Herisau, Switzerland). The latter measurement used 0.1 N NaOH to adjust to pH 8.2. Maturity indices and berry conditions are presented in Supplementary Table S27.

### Grape sampling for sensory analysis, volatile and non-volatile compounds, and RNA extraction

For each treatment, 20 berries were sampled from five clusters in each of the three biological replications. For sensory analysis, 50 berries were taken from each replicate and were mixed to create a pool of 150 berries for each treatment. Twenty-five berries from each replication were used for volatile-compound analysis for all treatments. Only treatments T0, TC, and T15 were analyzed for the presence of non-volatile compounds and were used for transcriptomic analyses. The berries were cut into quarters from the stem end to the stylar end, frozen in liquid nitrogen and kept at −80 °C pending processing, but not for more than 6 months. Prior to analysis, 10 quarter-berries were homogenized in an A11 grinder (IKA, Staufen, Germany) under liquid nitrogen for 20 s and the frozen powder was divided into subsets of 2 and 5 g, and of 1.2 g for the volatile and non-volatile analyses and RNA extraction, respectively.

### Sensory analysis

Grape tastings were conducted in two sessions: at harvest and after 6 weeks of storage followed by 48 h of shelf life at 20 °C. Each tasting panel of 11 trained panelists tasted five randomly selected berries from each treatment. Panelists were asked to evaluate the intensity score of the off-flavor in each sample by indicating: none = 0, low = 1, medium = 2, or high = 3.

### Volatile compound analysis

Crushed tissue powder (2 g) was immediately transferred to 20-ml amber vials (LaPhaPack, Langerwehe, Germany) that contained 1 g of NaCl (Merck, Darmstadt, Germany) and 2 ml of 20% (w/v) NaCl solution. A 50-µl aliquot containing internal standard 2-octanol (Sigma-Aldrich, St. Louis, MO, USA) at 10 mg/l was added to the vials, which then were sealed with magnetic screw caps fitted with pre-cut silicone white/polytetrafluoroethylene red LaPhaPack septa (Thermo Fisher Scientific, Waltham, MA, USA). GC/MS analysis was performed as previously described^86^.

### Analysis of non-volatiles

Each 5-g sample was lyophilized and maintained at 4 °C under dry nitrogen pending analysis. Before analysis, the sample was solubilized with 5 ml of H_2_O/CH_2_OH 50:50 (v/v) solution, and 100 µl of 4′,5′,7′-trihydroxy flavanone at 52 mg/l was added as an internal standard (Sigma-Aldrich, Milan, Italy). The samples were homogenized with an Ultra-Turrax (IKA) at 10,000 rpm for 30 s and then extracted in an orbital shaker at 200 rpm for 15 min at room temperature. After centrifugation at 7000 × *g* for 15 min the supernatant was filtered through a GHP 0.2-µm Acrodisc syringe filter (Pall, MI, USA**)** and collected in a vial for further chromatographic analysis.

LC/MS analyses were performed as previously described^87^ in an UHPLC 1290 Infinity apparatus (Agilent Technologies, Santa Clara, CA USA) coupled to a Model 1290 Infinity Autosampler (G4226A) (Agilent) and a Model 6540 accurate-mass Q-TOF mass spectrometer (Agilent) with a jet stream ionization source. For each sample, two analytical repetitions in negative ionization mode and full-scan acquisition were performed. A blank was run between successive samples to avoid cross-contamination. We used Agilent’s MassHunter data-acquisition software, version B.04.00 (B4033.2). We performed chromatography with a Zorbax reverse-phase column (RRHD SB-C18; 3 × 150 mm, 1.8 μm). The mobile phase comprised 0.1% (v/v) aqueous formic acid (A) and 0.1% formic acid in acetonitrile (B). The elution gradient program was as follows: 5% B isocratic for 8 min; from 5% to 45% B over 10 min; from 45% to 65% B over 5 min; from 65% to 90% B over 4 min; 90% B isocratic for 10 min. The flow rate was adjusted to 0.4 ml/min, column temperature was 35 °C, and injected sample volume was 5 μl. The Q-TOF conditions were: sheath gas nitrogen 10 l/min at 400 °C; drying gas nitrogen 8 l/min at 350 °C; nebulizer pressure 60 psi; nozzle voltage 0 kV; capillary voltage 3.5 kV. Signals in the *m/z* range of 100–1700, were recorded. Negative mass calibration was performed with a G1969–85000 standard mass mix (Supelco Inc., Bellefonte, PA, USA) and had a residual error for the expected masses of ±0.2 ppm. Lock masses were: TFA (trifluoroacetic acid) anion at *m/z* 112.9856 and HP-0921 (+formate) at *m/z* 966.0007 in negative-ion mode. MS/MS conditions were: collision energies between 20 and 60 eV to fragment the parent ions in the *m/z* range of 100–1700; acquisition rate – 2 spectra/s. Data processing was performed with Agilent’s MassHunter qualitative analysis software version B.05.00 (5.0.519.0). Confidence of compound identification based on accurate mass and isotope pattern was expressed as the “overall identification score”, computed as a weighted average of the isotopic pattern signals of the compound, such as exact masses, relative abundances, and *m/z* distances (spacing). The weights of these parameters were: W_mass_ = 100, W_abundance_ = 60, W_spacing_ = 50, mass expected data variation 2.0 mDa + 5.6 ppm, mass isotope abundance 7.5%, mass isotope grouping peak spacing tolerance 0.0025 *m/z* + 7.0 ppm. Target identification of compounds was performed with the home-made database of grape and wine metabolites called *GrapeMetabolomics*^88^.

### RNA extraction and analysis

Total RNA was extracted from 1.2 g of frozen berry powder^89^. 200 µl of extracted RNA was added 12 µl of DNAse buffer and 8 µl of 5-U/µl DNase TaKaRa DNase I kit (Cat#, 2270 A; TaKaRa, Japan). The mixture was incubated for 15 min at 37 °C. Two-hundred microliters of 24:1 (v/v) chloroform:isoamyl alcohol mixture was added to the samples and vortexed for 5 s. The mixture was then centrifuged at 20,000 × *g* for 5 min at 4 °C. The supernatant was precipitated in 1/3 volume of 10 M ammonium acetate and 10 volumes of 100% cold EtOH (−20 °C). The mixture was then incubated for 3 h at −80 °C and then centrifuged as described above for 30 min. The pellet was washed with 100 µl of 70% cold EtOH (−20 °C), which then was centrifuged as described above for 5 min. The EtOH was discarded and the pellet was left on the bench to dry for 15 min. The pellet was then placed in 30 µl of DEPC water and mixed by pipetting. The RNA was purified with an RNA Clean and Concentrator Kit (Zymo Research, Irvine, CA, USA).

### Bioinformatics analysis of RNA sequences

RNA was sequenced by the Technion Genome Center (Haifa, Israel) following integrity analysis with the TapeStation (Agilent), and sequencing libraries were generated with the TrueSeq RNA Sample Prep Kit v2 (Illumina) according to the manufacturer’s protocol. RNA sequencing was conducted with the Hiseq2500 (Illumina) applied to 60-bases-long single-end reads; we used V4 reagents (Illumina) that yielded 20–25 M pair reads per sample. The raw reads of nine libraries — three biological replications for each of the three treatments, T0, TC and T15 — were subjected to quality trimming and filtering and adapter removal by trimmomatic software^90^. Cleaned sequences were mapped to the latest reference genome version V2.1 of *V. vinifera* downloaded from the Cribi database^91^. The Bowtie software alignment protocol^92^ was used to align clean reads to the reference genome. Abundances were estimated for each *V. vinifera* transcript with the RSEM software package^93^. Bioconductor EdgeR^94^ of the Bioconductor R packages^95^ was used to identify differentially expressed transcripts for each biological replicate, based on the count estimates for each transcript. Transcripts were regarded as differentially expressed if they reached the threshold of FDR < 0.05 and the log of the proportional change was smaller than −1.5 or greater than 1.5. Transcript expression was normalized by using trimmed mean of M-values (TMM)^96^. All data analyzed in this manuscript were deposited in GenBank under bioproject accession number PRJNA386228, and the Raw Data SRA accession no. is SRP107049. Reproducibility of the replicates can be visualized by heat-map analysis (Supplementary Fig. S2). Grape gene orthologs related to specific metabolic stages were annotated based on MetGenMap^97^, Blast2Go^98^, Cribi^91^ data base (http://genomes.cribi.unipd.it) and on orthologous genes from tomato or Arabidopsis. *STS*, *WRKY* and *MYB* genes were annotated based on recent publication of the grape genome^18,21,22,69,99,100^. The GO term-enrichment analysis was performed based on the free web-based PANTHER system on Aug. 31, 2018^101^.

### Statistical analysis

Metabolite results were subjected to one-way ANOVA with Tukey’s honest significant difference (HSD) test for the non-volatiles or the nonparametric comparison with controls by using Dunn’s method for joint ranking for the volatiles on a JMP 13.0 platform (SAS Institute, Cary, NC, USA). Mosaic plot, PLS-DA and correlation coefficient for volatile compound analysis were based on the web-based MetaboAnalyst, version 3.0 (http://www.metaboanalyst.ca, accessed 26 Jan 2018)^102^. For the genes’ heat-map (Figs 2 and 4), genes TMM reads were centered by mean and normalized, and then subjected to gene cluster (centroid linkage) by Cluster 3.0^103^. Heat-maps were visualized based on and Java Treeview (http://jtreeview.sourceforge.net/). The Venn diagram was visualized based on online Venny V.2.1^104^. Molecular function visualization was performed on agriGO V.1.2^105^.

## Supplementary information

Supplementary Figures 1-4

Supplemetary Tables 1-27
