# Supplementary material for: Metabolomic and transcriptomic changes underlying cold and anaerobic stresses after storage of table grapes
Source: Sci Rep. 2019 Feb 27;9:2917. doi: 10.1038/s41598-019-39253-8 (PMC6393478; doi:10.1038/s41598-019-39253-8)

**Metabolomic and transcriptomic changes underlying cold and anaerobic stresses after storage of table grapes Itay Maoz**^1,2,3^, **Mirko De Rosso**^4^, **Tatiana Kaplunov**^1^, **Antonio Dalla Vedova**^4^, **Noa Sela**^5^, **Riccardo Flamini**^4^, **Efraim Lewinsohn**^2^ and **Amnon Lichter**^1,^*

Supplementary Figure 1 **-** Partial least squares Discriminant Analysis (PLS-DA) results of volatile compounds between A) five treatments separated or b) T0, Tc,T5 and T10 as compared to T15


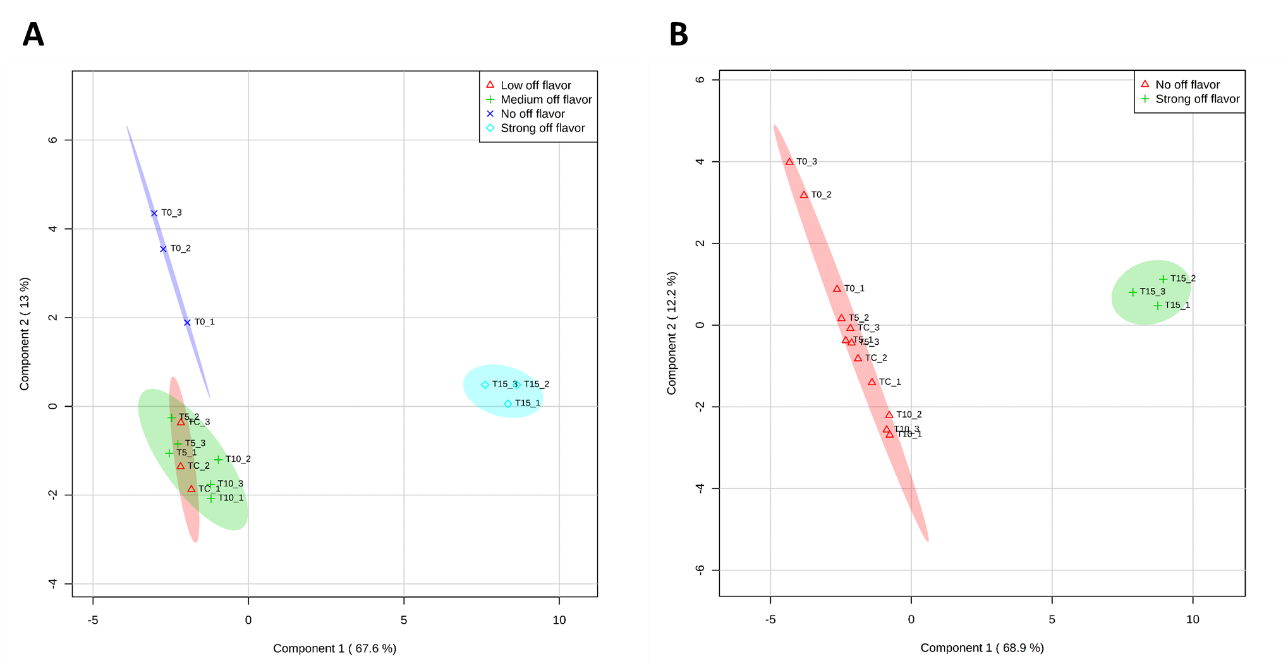


Supplementary Figure 2 **-** Heat-map analysis of the transcriptome data from T0, TC and T15.


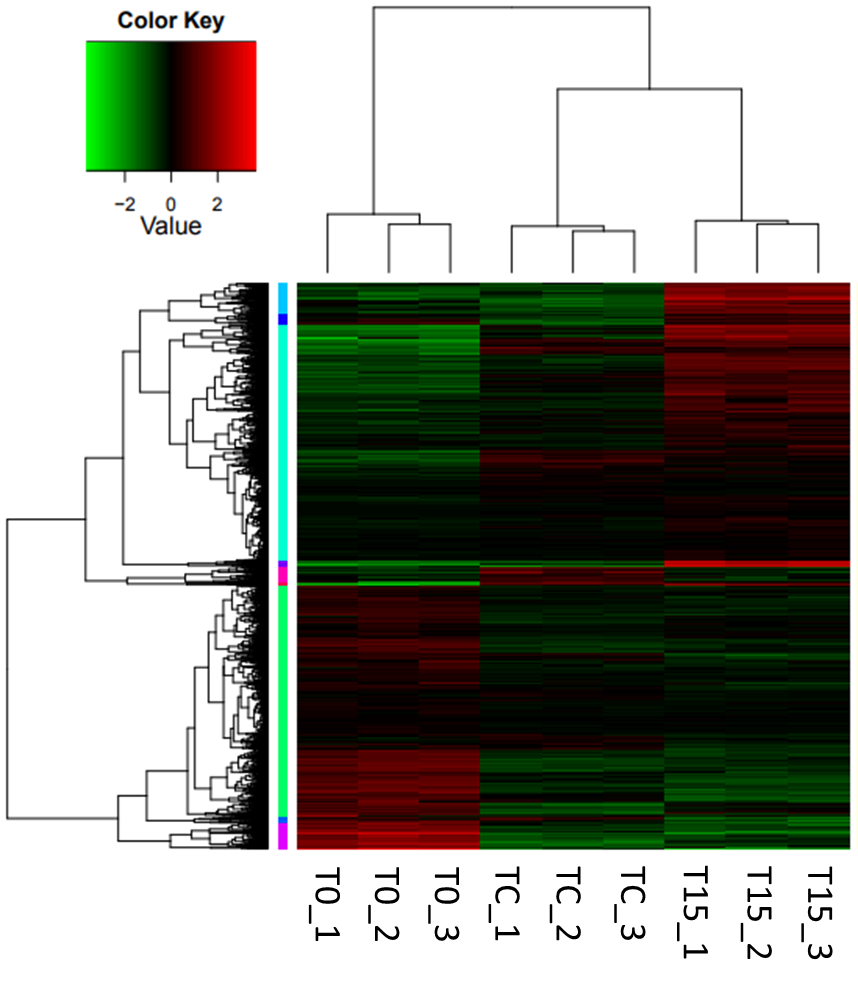


Supplementary Figure 3 - Venn diagram shows the upregulation and downregulation of genes between the 3 treatments. Grape berries of ‘Superior Seedless’ after harvest (T0), after storage for 6 weeks at low temperature (TC) or after storage at low temperature under O_2_ level of 5 kPa and CO_2_ levels of 15 kPa (T15). Only genes with at least one of the gene upregulated with LogFC>abs(1.5) and FDR<0.05. Genes were categorized as anaerobic-related if they were upregulated in T15 but not at TC and T0. Genes were categorized as cold stress-related if they were upregulated in both T15 and TC as compared to T0.


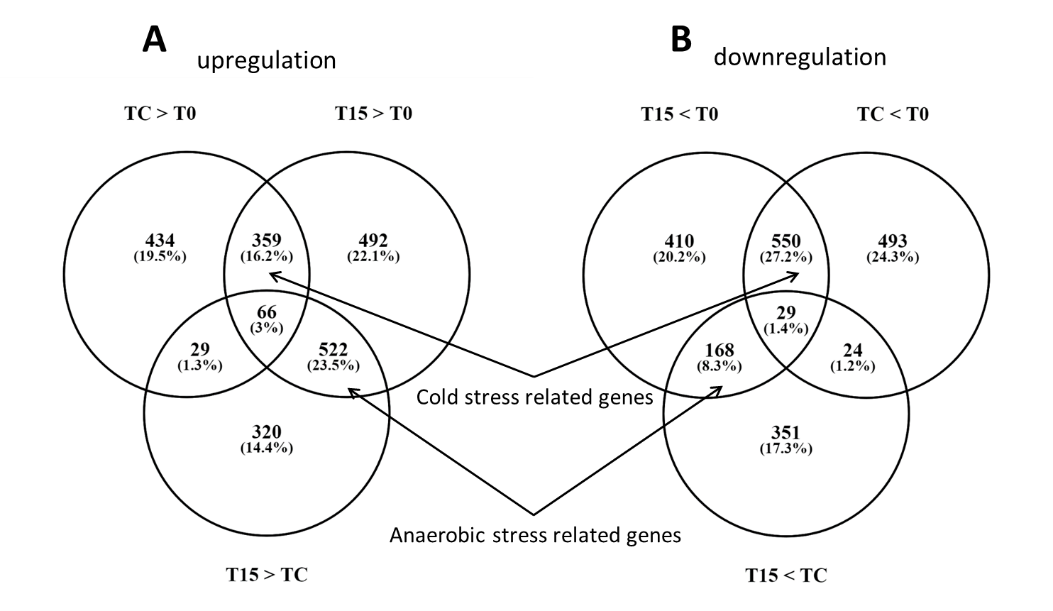


Supplementary Figure 4 - Changes in molecular function by upregulated genes under anaerobic conditions. Molecular function visualization was performed on agriGO V.1.2.


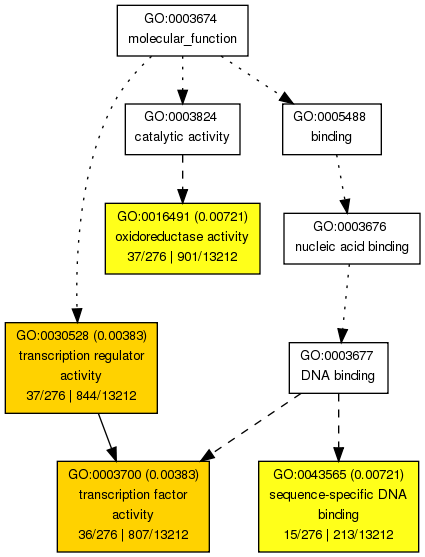

Supplement: Supplementary file 1 — Supplementary Figures 1-4 [file 41598_2019_39253_MOESM1_ESM.docx]
